# Design and Optimization Strategies for Flexible Quasi-Solid-State Thermo-Electrochemical Cells

**DOI:** 10.3390/ma16196574

**Published:** 2023-10-06

**Authors:** Bingchen Huo, Fengxia Kuang, Cun-Yue Guo

**Affiliations:** 1 School of Chemical Sciences, University of Chinese Academy of Sciences, Beijing 100049, China; huobingchen17@mails.ucas.ac.cn; 2 High & New Technology Research Center, Henan Academy of Sciences, Zhengzhou 450003, China; 3 Guangzhou Health Science College, Guangzhou 510925, China; kuangfengxian@126.com

**Keywords:** thermo-electrochemical cells, redox couples, quasi-solid-state electrolyte, electrode, device integration

## Abstract

Currently, efficient utilization of low-grade thermal energy is a great challenge. Thermoelectricity is an extremely promising method of generating electrical energy from temperature differences. As a green energy conversion technology, thermo-electrochemical cells (TECs) have attracted much attention in recent years for their ability to convert thermal energy directly into electricity with high thermal power. Within TECs, anions and cations gain and lose electrons, respectively, at the electrodes, using the potential difference between the hot and cold terminals of the electrodes by redox couples. Additionally, the anions and cations therein are constantly circulating and mobile via concentration diffusion and thermal diffusion, providing an uninterrupted supply of power to the exterior. This review article focuses mainly on the operation of TECs and recent advances in redox couples, electrolytes, and electrodes. The outlook for optimization strategies regarding TECs is also outlined in this paper.

## 1. Introduction

Effective utilization of low-grade thermal energy is currently a great challenge. Thermoelectric (TE) effects are an extremely promising way of generating electrical power from a temperature difference. Being noiseless and emission-free is the core advantages of thermoelectric conversion technology. Moreover, a complete thermoelectric device consisting of simply a thermoelectric material and electrodes can be integrated by circuit design, which can be applied to small electronic and wearable devices, allowing for the collection and utilization of thermal energy over a wider range of temperature differences. Thermoelectric materials are categorized into electronic thermoelectric materials, ionic thermoelectric materials, and thermo-electrochemical cells (TECs), based on their mechanism of operation [1,2,3,4].

**Figure 1:**
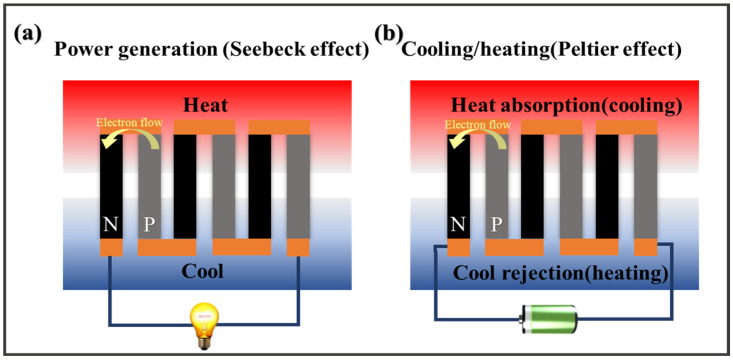
Schemes of thermoelectric device for power generation (**a**) and cooling (**b**).

The majority of research has focused on electronic thermoelectric materials, which are categorized as p-type or n-type depending on whether the carriers are holes or electrons. However, the Seebeck coefficient (*S*) of the electronic thermoelectric materials remains on the scale of microvolts per Kelvin, and several hundred pairs of p-n legs need to be connected in series to make the devices have a voltage output that is adequately high for real application (Figure 1) [5,6,7,8].

**Figure 2:**
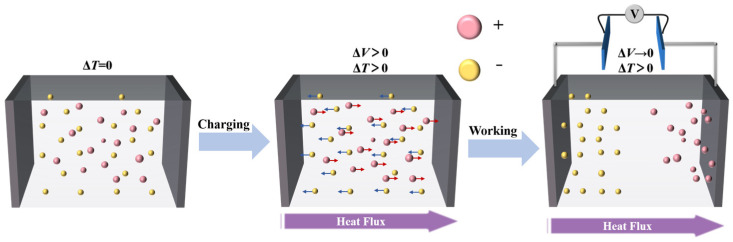
Schemes of ionic thermoelectric materials.

Ionic thermoelectric materials are based on the Soret effect, which involves an uneven distribution of ions in a conductor due to a temperature gradient. The difference in migration rates of anions and cations leads to a different stacking of ions at the hot and cold terminations, resulting in a potential difference between the two electrodes. However, ions cannot enter the external circuit and only depend on the accumulation of ions to generate an induced electromagnetic potential; therefore, the operation principle of ionic thermoelectric devices is similar to a capacitor, which cannot directly supply power to the outside world and can store thermal energy for converting to electrical energy (Figure 2) [9,10,11,12].

Like metal-ion batteries, TECs use a redox couple contained in an electrolyte. When a temperature difference occurs between two electrodes, the anion and cation at the electrode gain and lose electrons, respectively. Additionally, the internal anion and cation migrate via the concentration difference diffusion and thermal diffusion constantly in a cyclic manner, thereby generating uninterrupted electricity supply to the exterior [13,14,15]. The Seebeck coefficients of TECs tend to be >1000 μV K^−1^, two orders of magnitude higher than those of electronic thermoelectric materials. Meanwhile, the reaction inside the TECs is usually carried out in the solution system, which has the advantages of low cost, easy fabrication, etc. With the unique advantages of TECs, the optimization methods are gradually being diversified as the research on TECs progresses and the understanding of the principle of operation of thermoelectric batteries is deepened accordingly [16,17]. However, the present energy conversion efficiencies of TECs are not satisfactory; therefore, the optimization of the TECs for further increasing the thermoelectric conversion efficiency remains necessary.

Electrolyte, electrode, and mechanism layout are critical for optimizing TECs [18,19]. The optimization of electrode materials and the tuning of redox ions and electrolyte solvents have been focused on the pursuit of high thermoelectric conversion efficiencies. However, traditional liquid electrolytes suffer from complicated encapsulation and integration problems for wearable applications. To circumvent these issues, a possible approach is to consolidate the liquid electrolyte into a quasi-solid hydrogel electrolyte [20,21]. Unfortunately, there have been relatively few studies in this field, and this article reviews the advancement of gel-based TECs in recent years. Meanwhile, some problems and challenges are pointed out, and some new perspectives are offered for future research.

## 2. Wearable Thermo-Electrochemical Cells

### 2.1. Thermo-Electrochemical Cells

**Figure 3:**
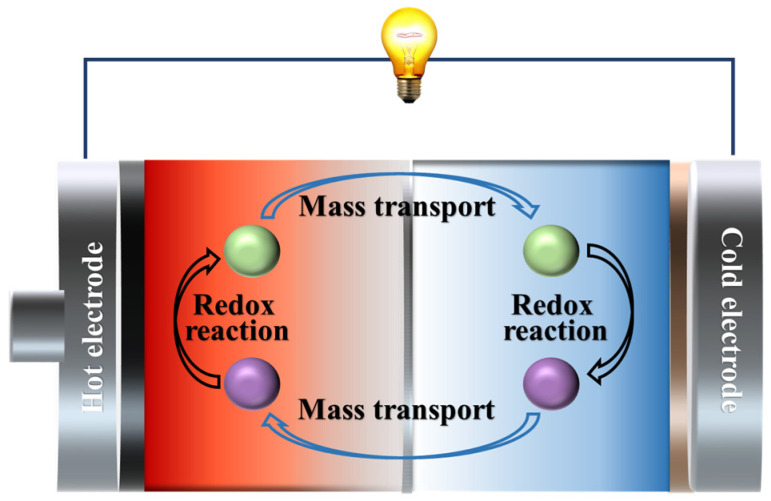
Schematic diagram of TECs.

TECs are constructed with electrolytes containing a redox couple with two electrodes and connected via an external circuit (Figure 3). A temperature gradient is applied to the electrode terminals with a changed electrode potential for redox ions due to the change in temperature. The high-potential electrode is the anode where oxidation reaction occurs, providing electrons to the external circuit; the low-potential electrode is the cathode where reduction reaction occurs, obtaining electrons from the external circuit. The valence state of ions changes at the electrode, which results in the formation of a concentration difference between the two terminals of the electrode, and the ions continuously migrate within the electrolyte through concentration difference diffusion and thermal diffusion, thus making the redox reaction continue and maintaining a consistent output of current and voltage. The operation of TECs involves two critical processes: (1) redox reactions at the electrodes and (2) ion transport processes in the electrolyte. In particular, the redox reaction at the electrode is associated with the Seebeck coefficient of TECs, and the ion transport in the electrolyte is associated with the conductivity and thermal conductivity of TECs [22,23].

#### 2.1.1. The Seebeck Coefficient of TECs

With the given temperature difference, the magnitude of the potential difference capable of being generated is one of the dominant factors in the energy conversion efficiency of TECs, i.e., the Seebeck coefficient.

(1) $$S=\frac{∆V_{OC}}{∆T}$$

In Formula (1), Δ*V*_OC_ is the open-circuit voltage and Δ*T* is the temperature difference between the two electrode terminals. This is the definition equation for the Seebeck coefficient of a thermoelectric material which applies to any thermoelectric material. The thermochemical effects in TECs can be described through redox reactions [24,25]:

(2) $$A+ne^{-}\to B\;$$

The Seebeck coefficient (*S*_e_) of the TECs is defined as:

(3) $$S_{e}={\left(\frac{\partial E}{\partial T}\right)}_{t=\infty }$$

In Formula (3), *E* is the electrode potential, which can be calculated using the Nernst equation, and *T* is the temperature. If the electrolyte is homogeneous internally, there exists [16,19,25,26]:

(4) $$S_{e}={\left(\frac{\partial E}{\partial T}\right)}_{t=\infty }=\left(\frac{1}{nF}\right)\times [\left(S_{B}+{\hat{S}}_{B}\right)-\left(S_{A}+{\hat{S}}_{A}\right)-n{\overset{̿}{S}}_{e}]$$

In Formula (4), *n* is the number of charges transferred in the redox reaction, F is Faraday’s constant, and *S*_A_ and *S*_B_ are the partial molal entropy of the ions, which result from taking one partial derivation of the total entropy of the ions with respect to the molar quantity of the ions. *Ŝ*_A_ and *Ŝ*_B_ are Eastman entropy, primarily derived from the interaction of the ions and their solvated shell structures with the surrounding solvent molecules as they move, which would be ignored in the majority of solutions. ${\overset{̿}{S}}_{e}$ is the transport entropy of electrons in the external circuit and usually only at the order of microvolts per Kelvin which is similarly negligible. Therefore, Equation (4) can be simplified to:

(5) $$S_{e}={\left(\frac{\partial E}{\partial T}\right)}_{t=\infty }\approx \frac{S_{B}-S_{A}}{nF}\;$$

In Formula (5), when the partial molar entropy of the reduced ions is greater than that of the oxidized ions, the Seebeck value is positive for the p-type TECs’ ion couple, whereas the partial molar entropy of the reduced ions is less than that of the oxidized ions when the Seebeck coefficient is negative for the n-type TEC’s ion couple [27].

#### 2.1.2. Performance Index of TECs

During TECs’ operation, the Seebeck coefficient is not the only determinant of device performance; the electrical conductivity (*σ*) and thermal conductivity (*κ*) of a unit cell deserve to be considered in equal measure. Three primary sources of overpotentials exist in TECs: (1) ohmic overpotentials, which are mainly caused by the internal resistance of the cell itself, the electrode resistance, and the circuit resistance. (2) Charge transfer overpotentials, which are related to the kinetics of redox charge transfer at the electrode surfaces. (3) Mass transfer overpotentials, which are related to the rate of movement of ions through the electrolyte and which encompasses diffusion of the ions, migration, and convection of the overall solution. Combined, these three factors influence the conductivity of the cell device [28].

The thermal conductivity of an electrolyte is also an important factor in the performance of TECs. For the liquid electrolyte, the thermal conductivity of the solution, convection, heat transfer, and ion mobility are all factors that influence the overall thermal conductivity. If the thermal conductivity is too high, the temperature gradient cannot be maintained between the two electrode terminals, resulting in a decrease in the temperature difference, a decrease in the voltage and current output, and, eventually, a temperature equilibrium TEC ceases to operate. Therefore, the performance of thermoelectric materials usually is evaluated by a dimensionless parameter, the thermoelectric merit value *ZT* [29].

(6) $$ZT=\frac{S^{2}\sigma }{\kappa }T\;$$

Similarly, thermoelectric device performance may be evaluated by the energy conversion efficiency *η*:

(7) $$\eta =\frac{P_{out}}{Q_{h}}$$

In Formula (7), *P*_out_ is the output power of the device and *Q*_h_ is the thermal energy supplied by the hot terminal. The output power is parabolic to the magnitude of the load resistance of the external circuit, and the output power reaches the maximum when the battery resistance is equal to the load resistance. The maximum energy conversion efficiency *η*_max_ can be calculated from the *ZT* value:

(8) $$\eta_{max}=\frac{T_{hot}-T_{cold}}{T_{hot}}\cdot \frac{\sqrt{1+ZT}-1}{\sqrt{1+ZT}+\frac{T_{cold}}{T_{hot}}}$$

In Equation (8), *T*_hot_ is the temperature of the hot terminal of the device, *T*_cold_ is the temperature of the cold terminal of the device, and the temperature adopted in the calculation of *ZT* value is the average temperature of the hot and cold terminals. Thermoelectric devices are still heat engines, essentially, and their maximum energy conversion efficiency is limited by the Carnot efficiency [26].

### 2.2. Electrochemical Thermogalvanic Effect

The electrochemical thermogalvanic effect consists of two main processes: (1) the oxidation-reduction reaction occurring at the electrode surface and (2) the electrolyte migration. The conversion efficiency of a thermo-electrochemical cell is intimately related to the *ZT* value; hence, the Seebeck coefficient and electrical conductivity can be increased, or the thermal conductivity can be decreased according to Equation (6). The magnitude of *S* is determined by the thermal power of the redox couple of materials in the electrolyte; the magnitude of *σ* is dependent on the resistance of the redox reaction occurring at the electrode surface and the transport resistance of the electrolyte, whereas the magnitude of *κ* is related to both the thermal conductivity in the presence of a temperature difference and the convection of the electrolyte [27,30].

*S* is dependent on the solvation-structure entropy difference (∆*S*) and concentration difference (∆*C*_r_) between redox substances. The absolute value of the charge of the redox substance in the electrolyte and the type of solvent and solute surrounding it deeply affect the magnitude of ∆*S*. Among the studies in the liquid thermocells system, [Fe(CN)_6_]^3−^/[Fe(CN)_6_]^4−^, Fe^2+^/Fe^3+^, and I^−^/I_3_^−^ are the most interesting. In general, redox couple with large absolute charge values and complicated complex structures possesses large ∆*S* [28,31,32].

**Figure 4:**
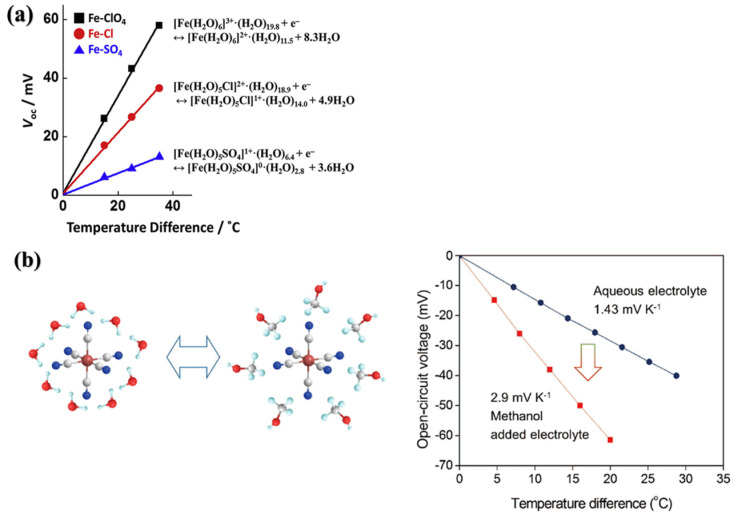
The voltage of different ferric salts at a range of temperature differences (**a**). Adapted from ref. [33] with permission. Copyright 2020, Elsevier. The Seebeck coefficient before and after methanol rearrangement of [Fe(CN)_6_]^4^− solvent shell and the voltage at different temperature with methanol (**b**). Adapted from ref. [34] with permission. Copyright 2017, Elsevier.

For Fe^2+/3+^ redox ions, their anions with different coordination sites have a bigger effect on the Seebeck coefficient. Kyunggu et al. have specifically investigated the effect of anions on the Seebeck coefficient of three common iron salts: Fe_2_(SO_4_)_3_/FeSO_4_, FeCl_3_/FeCl_2_, and Fe_2_(ClO_4_)_3_/FeClO_4_. The excellent performance of Fe^2+/3+^ perchlorate is attributed to the uncoordinated nature of its perchlorate anion, which inhibits the reduction of *S* and prevents the formation of ionic couples (Figure 4a shows the voltage of different ferric salts at various temperature differences.) [33]. [Fe(CN)_6_]^3−^/[Fe(CN)_6_]^4−^ is the redox couple that has achieved the highest thermal power to date, and it remains possible to alter the solvent environment of the ions to increase the Seebeck coefficient. Kim et al. reported that with the addition of an organic solvent with appropriate solubility parameters to the aqueous electrolyte of [Fe(CN)_6_]^3−^/[Fe(CN)_6_]^4−^, the electrochemical thermopower can be more than doubled to 2.9 mV K^−1^. The addition organic solvent results in a noticeable rearrangement of the solvation shells which, in turn, leads to an increase in the entropy change of the whole redox system, thereby increasing the electrochemical thermopower (Figure 4b shows the Seebeck coefficient before and after methanol rearrangement of [Fe(CN)_6_]^4−^ solvent shell and the voltage at different temperature with methanol.) [34]. Prediction of heat power (i.e., thermoelectric temperature coefficient) with molecular dynamics simulations could allow for simpler and more convenient optimization of redox couples. Chen et al. noticed the *S* of Fe^2+/3+^ can reach 3.8 ± 0.5 mV K^−1^ in a mixture of acetone–water solvent with molecular dynamics simulation, which matches the experimental value. The discovery provided insight into the design of solvation shell sequences to develop electrolytes with high *S.* Apart from changing the solvent environment, the addition of other additives which modify the redox ion hydration shell to optimize the Seebeck coefficients is commonly employed [35]. Duan et al. introduced guanidine salt with high ionic sequence and amide derivative urea with high polarity into Fe(CN)_6_^4−/3−^ aqueous solution, and their synergistic effect resulted in the enhancement of the Seebeck coefficient of Fe(CN)_6_^4−/3−^ from 1.4 mV K^−1^ to 4.2 mV K^−1^ and the growth of power density from 0.4 mW K^−2^ m^−2^ to 1.1 mW K^−2^ m^−2^. Guanidine salts are one of the highest cationic salts in the chaotropic sequence which can destabilize non-covalent bonding forces or destroy the structure of macromolecular proteins [36].

**Figure 5:**
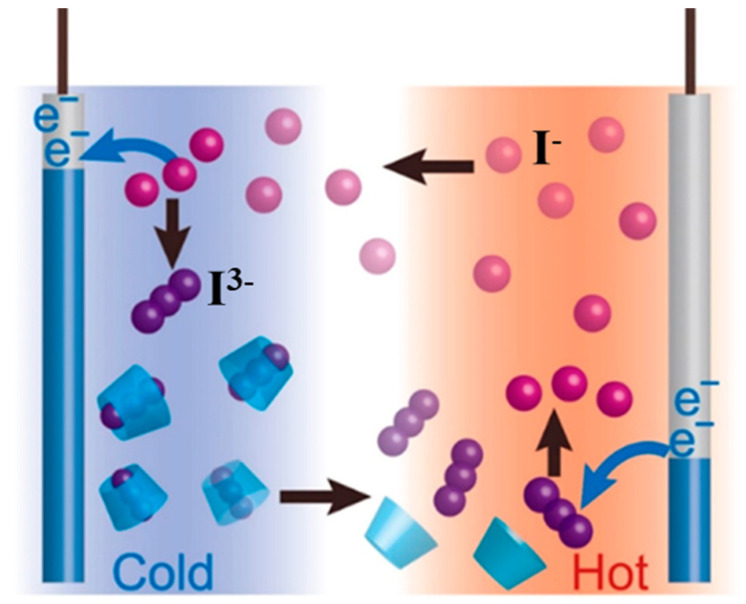
Schematic of supramolecular thermocell reaction of α-CD and I^3−^/I^−^ redox pair. Adapted from ref. [38] with permission. Copyright 2016, American Chemical Society.

In addition to increasing ∆*S*, an alternate way to increase *S*_e_ is to increase ∆*C*_r_ [37]. However, redox couples cannot permanently maintain a state of concentration difference between the hot and cold ends. Because the concentration difference state is unstable from a thermodynamic perspective, it spontaneously decays to a homogeneous state. The ∆*C*_r_ equals zero while the electrolyte is in a stable state. Zhou et al. exploited the temperature-sensitive properties of cyclodextrins and the host-guest interaction with I^3−^ to create an I^−^/I^3−^ concentration difference between the hot and cold ends, resulting in an increase in the Seebeck coefficient from 0.86 mV K^−1^ to 1.97 mV K^−1^. Figure 5 shows that at the cold terminal, the hydrophobic property of the inner ring of α-CD is exploited to form an α-CD-I^3−^ complex by combining with the similarly hydrophobic I^3−^, which prevents I^3−^ ions from participating in the reaction and decreases the concentration at the cold terminal. However, the α-CD-I^3−^ composite has a temperature-sensitive property and releases I^3−^ ions upon dissolution at the hot terminal, consequently resulting in a different concentration level of I^3−^ at the hot and cold terminals, increasing the Seebeck coefficients [38]. Yu et al. employed guanidine salts and Fe(CN)_6_^4−^ to form thermosensitive crystals that reduced the concentration of Fe(CN)_6_^4−^ at the cold terminal and resolved at the hot terminal, with no effect on the rate of the redox reaction. This results in the formation of a continuous concentration gradient in the solution, which increases the Seebeck coefficient from 1.4 mV K^−1^ to 3.73 mV K^−1^. Meanwhile, the solid crystals formed also effectively suppress the thermal conductivity of the liquid and, ultimately, increase the relative Carnot efficiency to 11% [39]. Furthermore, concentration theory may also be applied to change the sign of the Seebeck coefficient, i.e., to change the type of reaction that occurs at the hot and cold terminals. Duan et al. achieved an increase in the absolute value of the Seebeck coefficient of the I^−^/I^3−^ ion pair and a change in the sign of the Seebeck coefficient from a p-type to an n-type thermocell via the incorporation of a temperature-sensitive nano-microgel (PNIPAM) into an aqueous solution of I^−^/I^3−^. PNIPAM has a hydrophilic to hydrophobic phase transition at around 32 °C, which also changes the gel polymer chain backbone from stretching to condensation and controls the equilibrium of the I^−^/I^3−^ redox reaction. The hydrophobic phase of PNIPAM dominates at the hot terminal, and the I^3−^ ion combines with PNIPAM due to the hydrophobic effect; thus, the concentration of I^3−^ ion decreases at the hot terminal, and the oxidation reaction of conversion from I^3−^ to I^−^ occurs at the hot terminal. The hydrophilic phase of PNIPAM dominates the backbone stretching at the cold terminal, and PNIPAM-I^3−^ releases I^3−^ ions, which leads to the reduction reaction of I^−^ to I^3−^ conversion at the cold terminal, thus changing the original I^−^/I^3−^ redox direction and altering the sign of the Seebeck coefficient. The concentration difference constructed in this manner resulted in a higher absolute Seebeck value, from 0.71 mV K^−1^ to −1.91 mV K^−1^ [40]. Concentration difference effects focus on regulating the Seebeck coefficient, which requires a specific ion in the redox couple to combine with the additive to form a temperature-sensitive substance in order to enable the formation of concentration difference effects of ions at the hot and cold terminals of the electrodes. This effect has a great ability to regulate the Seebeck coefficient and can also change the direction of the redox reaction; however, the resulting conjugates may influence the rate of the redox reaction, resulting in irreversible side-reactions during the thermal cell cycling, which causes a decrease in the cycling performance and, finally, an attenuation of the output power [27,41,42,43].

### 2.3. Quasi-Solid-State Electrolyte

In general, the conductivity of the liquid electrolyte is approximately three orders of magnitude higher than that of conventional solid state thermoelectric cells. However, thermal convection would be unavoidable in the mass transfer process in liquid electrolyte. Thermal convection reduces the thermal gradient between the electrodes, thereby reducing the Seebeck coefficient of TECs. Inhibiting thermal convection has become an effective method of optimizing the performance of TECs [44,45]. Zhang et al. achieved an improvement in the thermal gradient within the electrolyte through the application of an electrolyte-filled porous material (called a thermal separator) to the cold electrode of the hot cell. This new thermocell structure shifts the interelectrode temperature gradient in the electrolyte towards the cold electrode, thereby increasing the average ionic conductivity along the interelectrode ion path and increasing the temperature difference by ~20 °C, resulting in a high power density of ∼12 W m^–2^ [30]. However, the reduction of thermal convection in the liquid electrolyte is a complex and costly process. Fortunately, thermal convection in the gel electrolyte is attenuated, favoring the establishment and maintenance of a temperature gradient, thereby achieving higher energy conversion efficiencies. What is more, in the practical application of TECs, the leakage issue of liquid batteries and non-portability are significant hindrances to the process of adoption. And the gel electrolyte allows for arbitrary cutting and shaping, which essentially solves the leakage problem of liquid electrolyte solutions. Therefore, quasi-solid electrolytes are gradually attracting attention due to their advantages of self-encapsulation, leakage prevention, and flexible characteristics. Meanwhile, thermal convection is ignored in gel electrolytes by the property of the quasi-solid state; hence, thermal conduction becomes the dominant form of heat transfer in gel electrolytes, which reduces the thermal conductivity significantly [46,47,48,49,50]. The thermopower of several typical redox couples in gel electrolytes is presented in Table 1, including those examples reviewed above.

Due to the quasi-solid nature of the gel electrolyte, a solvation effect similar to that observed in liquid electrolytes is present. The solvation effect in gel electrolytes refers to the interaction between solvent molecules and electrolyte ions. When a solvent molecule approaches an electrolyte ion, it electrostatically attracts the ion, forming a solvation layer. This formation alters the movement characteristics of electrolyte ions and impacts the conductivity of the gel electrolyte.

Specific effects of solvation include (1) enhancement of ionic solubility (the formation of a solvation layer increases the solubility of electrolyte ions in solution, rendering them more soluble in solvents). (2) Slowing down ion migration (the presence of a sol ion movement results in slower migration rates for electrolyte ions within the gel electrolyte). (3) enhanced ion conduction (although individual ion migration is slowed by the presence of a solvation layer, it also increases). The formation of this layer enables ionic flow, thereby enhancing conductivity within the gel electrolyte.

Organic solvents can indeed provide a wider potential range or electrochemical window which can enable higher voltage cells. This can be an advantage over aqueous gel electrolytes, as it can potentially lead to higher energy densities. Furthermore, organic solvents can dissolve a wider range of materials, including many that cannot be dissolved in water, which offers more flexibility in terms of electrode material choice. However, the volatility of organic solvents can be a significant drawback, as it can lead to safety issues. Solvents can evaporate or leak, potentially causing harm to people or the environment. They can also contribute to the flammability of a battery system. Other challenges with organic solvents include their typically lower ionic conductivities compared to aqueous systems and their compatibility with other battery components. For instance, certain organic solvents can dissolve or swell certain plastics used in battery construction, leading to mechanical instability. In brief, whether organic solvents provide an “edge” over aqueous electrolytes depends on the specific requirements of the application, and a balance must be struck between performance, safety, and cost considerations [54,55,56,57].

PVA is a promising candidate for quasi-solid electrolyte substrates due to its biocompatibility, non-toxicity, non-corrosivity, and excellent water solubility. Zhou et al. synthesized a PVA-FeCl^2+/3+^ gel electrolyte with high mechanical strength and low charge transfer resistance, which uses HCl as a supporting electrolyte, with a *S*_e_ of 0.8 ± 0.02 mV K^−1^, current density of 16.1 A m^−2^, and power density of 63.7 mW m^−2^ at a Δ*T* of 20 K [58]. Liu et al. combined stretch-induced crystallization with the thermoelectric effect to present a high-strength quasi-solid stretchable PVA thermoelectric thermocouple (SPTC) with a tensile strength of 19 MPa and a thermopower of 6.5 mV K^−1^. The SPTC has a high tensile strength of 1300%, an ultra-high toughness of 163.4 MJ m^−3^, and an output power density of up to 1969 μW m^−2^ K^−2^ [59]. Gao et al. designed PVA anisotropic polymer networks to produce aligned channels for ionic conduction and hierarchically assembled crystalline nanoprotofibres for crack passivation. The ionic conductivity of the anisotropic thermocouples was increased by more than 400% and the power density was comparable to that recorded for state-of-the-art quasi-solid thermocouples. Furthermore, compared to available quasi-solid thermocouples with the best mechanical properties, the material achieves bionic strain stiffness, with toughness and strength improved by more than 1100% and 300%, respectively [60]. Employing PVA (a commercially available polymer) as a gel electrolyte is an economically effective approach that offers the possibility of large-scale integration of TECs in the future.

**Figure 6:**
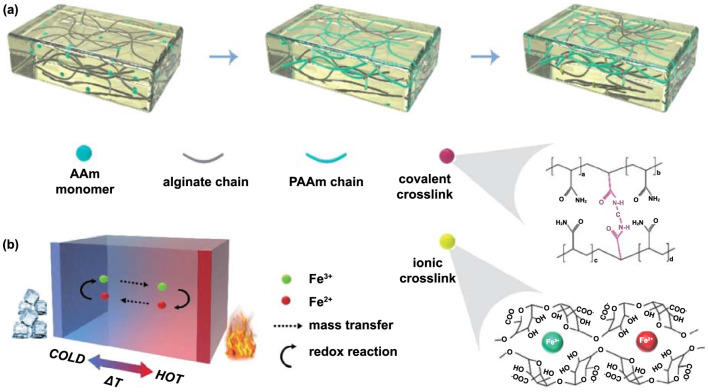
Illustration of the forming process of the covalently cross-linked network and the ionically cross-linked network within the hydrogel body. The molecular schematics reveal the structures of covalent and ionic cross-links (**a**). Illustration of the working mechanism of a TEC based on thermogalvanic effect (**b**). Adapted from ref. [64] with permission. Copyright 2022, Springer Nature.

In addition to PVA, gelatin, cellulose, agar, sodium polyacrylate, polyacrylamide, and other polymers are also promising materials for composing hydrogel matrices, which possess more abundant functional groups and may offer more possibilities for optimizing the performance of TECs. While the gels formed from these materials do not have the same electrical conductivity as the polyelectrolyte gels designed and synthesized, these materials will remain prospective considering the factors of industrialization. Currently, the majority of designed and synthesized polyelectrolytes are primarily utilized in lithium batteries, with limited research conducted on their application in thermal batteries [61,62]. The investigation of polyelectrolyte utilization in TECs holds significant value as it offers potential insights into the design and optimization of gel electrolytes. Zeng et al. fabricated a series of semi-interpenetrating network (semi-IPN) polymer electrolytes based on novel liquid crystals (C6M LCs) and poly(ethylene glycol) diglycidyl ether (PEGDE). The LCs dramatically improved not only the mechanical properties of the electrolyte membranes by constructing a network structure with PEGEDE but also created a stable ion transport channel for ionic conduction. This freestanding flexible SPE exhibits an excellent ionic conductivity (5.93 × 10^−5^ S cm^−1^ at 30 °C) with a very wide electrochemical stability window of 5.5 V [63]. The challenge, however, lies in the incorporation of cost-effective commercial polymers into TECs and enhancement of their performance. Chen et al. proposed a flexible quasi-solid-state TEC, via the rational design of a hydrogel electrolyte, which simultaneously modulates the thermoelectric effect and mechanical robustness by redox-coupled multivalent ions (Figure 6) [64]. The enhancement of the mechanical strength of the gel electrolyte establishes a solid foundation for the integration of more intricate wearable flexible electronic devices.

Chen et al. also introduced the high electrochemical potential of redox couple (Sn^4+^/Sn^2+^) into a flexible and stretchable (within a strain of 100%) composite hydrogel (polyacrylamide/acidified SWCNTs) and constructed a gel state with a large and stable *S*_e_ of 1.59 ± 0.07 mV K^−1^. The strain sensitivity originated from the well-dispersed acidified SWCNT network, and the polyacrylamide hydrogel matrix endows the TEC with an additional role as a self-powered strain sensor for monitoring various human motions relating to the finger, wrist, and elbow [65]. The introduction of redox couple not only provides the hydrogel with excellent thermoelectric conversion capability but also acts as an ionic cross-linking agent to generate double cross-linking structures, which result in the formation of reversible bonds for effective energy dissipation. With a high Seebeck coefficient of 1.43 mV K^−1^ and a significantly improved fracture toughness of 3555 J m^−2^, the optimized TECs are able to maintain stable thermos-electrochemical properties under various harsh mechanical stimuli [64]. Furthermore, Chen et al. designed an aqueous eutectic gel electrolyte based on a concentrated lithium bis(trifluoromethane) sulfonimide (LiTFSI) solution, which can be used to achieve freezing resistance and self-humidification capability by regulating the hydrophobicity in the hydrogel. It also exhibits long-term environmental stability without the requirement for encapsulation or packaging. Hydrogel electrolyte collision properties can inhibit ice crystallization, and molecular dynamics simulations suggest that the strong coordination effect between lithium ions and water molecules across a wide range of temperatures is an important potential mechanism [66]. As a result, to further advance the development of efficient design strategies for gel electrolytes in order to regulate and optimize their thermoelectric properties, two crucial issues need to be addressed. Firstly, it is necessary to manipulate the gel structure in order to modify the partial molar entropy of the redox reaction and achieve higher *S*_e_. Secondly, there is a need to establish a well-structured network within the gels that facilitates ion/charge transport, resulting in faster ion diffusion rates, reduced mass transfer resistance, and lower interfacial transfer resistance.

### 2.4. Electrode

In general, Pt has been employed as an electrode to maintain the simplicity and reverse ability of the redox reaction in the HCF electrolyte (hexacyanoferrates), however, Pt remains rare and expensive, which prevents the commercialization of TECs. Some non-precious metals, including copper, nickel, tungsten, and stainless steel, have also been utilized as materials for electrodes [19,32,67,68]. Carbon is a widely available material and therefore carbon electrodes have attracted widespread attention recently, especially nanostructured carbon materials which typically have high electrical conductivity, rapid redox kinetics, large electrochemically active surface area (ESA), and energetic behavior in relation to HCF redox couples [69,70,71,72]. However, the manufacturing process for these carbon-based electrodes is complicated, not to mention the inherent hydrophobicity which hinders ion transport and thus prevents further performance improvements. Hence, a simplification of the fabrication process of carbon-based electrodes is necessary to make them easily scalable and to improve their hydrophilicity for better penetration into the electrolyte [73].

MXenes, a two-dimensional transition metal carbide and nitride material, have emerged to provide a promising approach to the construction of high-performance TEC electrodes. The high conductivity and hydrophilicity of Mxenes allow for high electron transport speeds and mass transfer. Meanwhile, the reduction activity of the transition metal atoms on the surface is more favorable for electrochemical processes, with a large surface area providing a high ESA. The layered architecture of MXenes, which facilitates the insertion of molecules and ions, is beneficial for the regulation of properties and the assembly of multilayers [74,75,76,77].

Wei et al. constructed flexible thin-film electrodes with ternary composites of Ti_3_C_2_T*_x_*, polyaniline (PANI), and single-walled carbon nanotubes, which showed significantly enhanced thermo-electrochemical properties compared to the widely used precious metal platinum electrodes. A porous layered structure with a large electrochemically active surface area was formed in the ternary composite electrode. Results of experiments and simulations indicate that the synergetic effect of Ti_3_C_2_T*_x_* and PANI promotes the mass and charge transport at the electrolyte–electrode interface, generating a TEC with an output power of 13.15 µW cm^−2^ at a Δ*T* of 40 K. TEC can also respond rapidly to minute temperature differences between human bodies and the environment, indicating that it has great potential for harvesting low-grade heat to power small electronic devices [73].

## 3. Device Integration and Applications

In the application of TECs, the thermoelectric conversion efficiency is one of the standards for evaluating the performance of the device. The methods commonly employed to enhance the thermoelectric conversion efficiency of TECs include:

1. Optimizing thermoelectric materials: materials with high Seebeck coefficients and low thermal conductivity should be selected. A high Seebeck coefficient can increase the thermoelectric conversion efficiency, while a low thermal conductivity can reduce heat dissipation.
2. Increasing the temperature gradient: the thermoelectric conversion efficiency is directly proportional to the temperature gradient. It can improve the temperature gradient by increasing the temperature of the high-temperature heat source or decreasing the temperature of the low-temperature heat source, thus improving the thermoelectric conversion efficiency.
3. Reducing heat dissipation: optimization of the insulating material and structural design of the thermocells can reduce heat dissipation and improve the thermoelectric conversion efficiency.
4. Cascade thermoelectric module: multiple thermoelectric modules are cascaded together to improve the thermoelectric conversion efficiency. In the cascade thermoelectric module, the waste heat from the high-temperature heat source can be further utilized, thus improving the energy conversion efficiency of the whole system [2,19,27,78].

**Figure 7:**
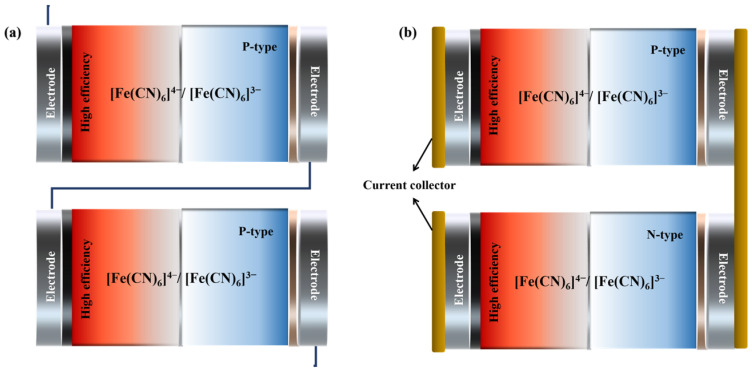
Schematic diagram of *Z*-shaped connection (**a**) and *Π*-shaped connection (**b**).

Besides enhancing the efficiency of a single cell, a further research focus is on device integration and applications. Enhancing the integration of thermal cells stands as a paramount strategy for bolstering the efficacy of thermoelectric conversion processes. The main integration methods are *Z*-shaped, a serrated connection of identical single cells in series, and *Π*-shaped, a combination of p-type and n-type cells in series (Figure 7). Thermocouples, where oxidation reaction occurs at the hot electrode, are typically defined as p-type, whereas thermocouples in which reduction reaction proceeds at the hot electrode are defined as n-type. In thermoelectric devices, *Π*-type and *Z*-type configurations are two prevalent connection modes. Comparatively, the *Π*-type connection is more efficient due to the following reasons:

1. Current distribution: in *Π*-type connections, the current distribution across the thermoelectric elements is more uniform, with each element carrying the same current. However, in *Z*-type connections, the current is distributed across different elements due to their series connection, resulting in an uneven current distribution that affects efficiency.
2. Thermal resistance: in *Π*-type connections, each thermoelectric element exhibits the same thermal resistance due to their parallel connection, thereby reducing the overall thermal resistance. In contrast, in *Z*-type connections, the thermal resistance increases due to the series connection of the thermoelectric elements, hence affecting their efficiency.
3. Voltage distribution: in *Π*-type connections, the voltage distribution is more uniform, with each element having the same voltage. However, in *Z*-type connections, the voltage is distributed across different elements due to their series connection, leading to an uneven voltage distribution that affects the efficiency.

Overall, the *Π*-type connection outperforms the *Z*-type connection in terms of current distribution, thermal resistance, and voltage distribution, making it more efficient. The *Π*-shaped connected device has the advantage of achieving maximum power output with a single state-of-the-art cell [1,40,47,79,80].

Shi et al. designed a fatigue-resistant and highly conductive hydrogel thermocouple with photothermal conversion capability for non-contact self-powered applications. At a temperature difference of 20 K, the output voltage rises from ≈0.05 V to 0.85 V when individual thermocouples are assembled into an array of 20 cells [21]. However, the device integration process using *Z*-shaped connections is complex, with a large contact resistance between the electrodes and the *Z*-shaped wires. The *Π*-shaped connection, on the other hand, simplifies the integration process and enables good contact between the electrodes and the collector. Xu et al. designed a p-n pair hydrogel electrolyte by choosing Fe(ClO_4_)_3_/Fe(ClO_4_)_2_ as the n-type ion couple. By integrating and fabricating a conformal portable thermal battery device, 14 pairs of p-n-connected cells achieved an output voltage of 0.16 V at Δ*T* = 4.1 K [81]. Unfortunately, integrated devices via *Π*-shaped connections remain inefficient due to a lack of high-performance n-type batteries.

## 4. Summary and Outlook

TECs represent an emerging technology for thermoelectric conversion which has attracted extensive attention from researchers in both academia and industries. Despite the significant advances in TECs which have been achieved via the optimization of electrolytes, electrodes, insulation materials, and device modules, numerous challenges remain to be overcome.

Regarding the electrolyte, the modification of existing redox pairs or the development of new ionic pairs, the alteration of the solvent environment and the addition of additives to form a concentration difference, or the destruction of the ionic solvent shells, in addition to the use of quasi-solid electrolytes, have all contributed to the great enhancement of the Seebeck coefficient of the thermal batteries or the output power. However, the following shortcomings still exist and require further development and optimization.

The interaction of redox ions with solvent molecules is one of the important factors determining the Seebeck coefficient. However, none of the relevant solvent parameters exhibit regularity in the effects on the ionic Seebeck coefficients; therefore, the specific mechanism of the solvent molecules’ influence on the redox ions has not been clarified, with a lack of exploration into the in-depth mechanism of exactly how the solvent affects the Seebeck coefficients.

Integrated device reliability is also one of the factors to be considered. In general, thermoelectric devices require the integration of multiple p-type and n-type TECs to obtain a stable voltage output. Although existing p-type TECs (e.g., the [Fe(CN)_6_]^3−^/[Fe(CN)_6_]^4−^ system) have been relatively sophisticated, the development of corresponding n-types are still unsatisfactory. Further research on high-performance n-type redox couple is critical for the optimization of devices for TECs. For example, perchlorate redox pair (Fe^2+^/Fe^3+^) exhibited a high Seebeck coefficient of 1.76 mV K^−1^ and high solubility (>1 M) in aqueous electrolytes [33,34]. Another focus is on the stability of TEC devices in extreme environments. The effects of cryogenic and high temperatures on material properties should be taken into account in the design of TECs, and the widening of operating temperature range of TECs, in particular, should be an additional focus.

The application of flexible thermoelectric devices for thermal batteries in wearable devices lacks extensive research, especially the adaptation of flexible electrodes and gel electrolytes and the improvement of electrodes for the qualities of gel electrolytes, while the comprehensive performance of thermoelectric devices after their integration remains to be explored.

## Figures and Tables

**Table 1:** Thermopower values for the typical redox couples in gel electrolytes.

Redox Couple	Matrix	Thermopower (mV K^−1^)
FeCN^4−/3−^	Gelatin	17 (Δ*T* = 7 K) [51]
FeCN^4−/3−^	Cellulose	14 (Δ*T* = 15 K) [46]
FeCN^4−/3−^	Poly(sodium acrylate)	−1.09 ± 0.04 (Δ*T* = 25 K) [48]
FeCN^4−/3−^	PVA	−1.21 (Δ*T* = 10 K) [47]
I^−^/I^3−^	*N*-isopropylacrylamide	−1.91 (Δ*T*= 10 K) [40]
[Co(bpy)_3_]^2+/3+^[NTf_2_^−^]_2/3_	Poly(vinylidene fluoride-*co*-hexafluoropropene) (PVDF-HFP)	1.56 ± 0.01 (Δ*T* = 15 K) [52]
Co(bpy)_3_]^2+/3+^[NTf_2_^−^]_2/3_	Polyvinylidene difluoride and 3-methoxypropionitrile (PVDF-MPN)	1.84 ± 0.01 [53]
